# Sex-Specific Diet–Microbiota Interactions in Ageing: Implications for Healthy Longevity

**DOI:** 10.3390/nu17243833

**Published:** 2025-12-08

**Authors:** Julieta Hernández-Acosta, Armando R. Tovar, Nimbe Torres

**Affiliations:** Departamento de Fisiología de la Nutrición, Instituto Nacional de Ciencias Médicas y Nutrición Salvador Zubirán, Vasco de Quiroga 15, Colonia Belisario Domínguez Secc XVI, Tlalpan, México City 14080, Mexico; julieta.hdez@hotmail.com (J.H.-A.); armando.tovarp@incmnsz.mx (A.R.T.)

**Keywords:** healthy ageing, sex differences, gut microbiota, dietary patterns, microbial metabolites, inflammageing, precision nutrition

## Abstract

**Background/Objectives**: Diet–microbiota interactions shape ageing; however, their sex-specific dimensions remain poorly defined. Human studies rarely stratify analyses by sex, while most evidence of sex-dependent microbial and metabolic responses comes from preclinical models. This review synthesizes current findings on the sex-specific pathways linking diet, microbiota, and healthy ageing. **Methods**: A narrative review was conducted by integrating human observational studies, randomized controlled trials, and mechanistic animal research. Evidence was organized into four domains: (1) age-related changes in gut microbial composition; (2) microbiota-derived metabolites; (3) dietary patterns and functional nutrients; and (4) sex-specific endocrine and immunometabolism interactions influenced by the gut microbiota. **Results**: Ageing is characterized by dysbiosis, loss of short-chain fatty acid (SCFA)-producing taxa, expansion of *Proteobacteria*, and reduced production of key metabolites including butyrate, indoles, and polyamines. Dietary fiber, polyphenols, omega-3 fatty acids, and plant-based proteins help restore these pathways and mitigate inflammaging. Sex differences persist into later life: women show reduced estrobolome activity and SCFA decline after menopause, whereas men display higher levels of pro-atherogenic metabolites such as trimethylamine N-oxide (TMAO). Nutritional interventions, probiotics, and microbial metabolites exhibit sex-dependent responses in both human and animal studies. **Conclusions**: Diet–microbiota interactions shape ageing outcomes through sex-specific metabolic, hormonal, and immunological pathways. Incorporating sex as a biological variable is essential for developing personalized, nutrition-based strategies to support healthy ageing.

## 1. Introduction

The global increase in life expectancy underscores the need to identify the biological determinants that support healthy ageing [1]. Among these, the gut microbiota has emerged as a central regulator of host physiology influencing metabolic, immune, and neurocognitive functions throughout life [2]. Diet exerts a major influence on the composition and activity of the gut microbiota [3], thereby opening pathways for nutritional interventions aimed at promoting healthy ageing through modulation of the gut ecosystem. Moreover, recent evidence suggests that biological sex introduces an additional layer of variability in the ageing-related process [4]. Collectively, the interplay between diet, microbiota, and sex represents a promising yet underexplored frontier in ageing research.

Throughout life, the gut microbiota undergoes continuous remodeling driven by diet, lifestyle, and intrinsic ageing processes [5,6]. However, the extent to which these microbial trajectories are modulated by sex remains uncertain. Growing evidence indicates that microbial metabolites, such as short-chain fatty acids (SCFAs), bile acids, polyamines, and indole derivatives, act as molecular intermediaries linking microbial activity to host metabolic and immune regulation during ageing [7,8,9]. Their production is modulated not only by diet and age, but also by sex-related factors such as the hormonal environment [10]. Studies in adults and older individuals show that bile acids profiles and several indole metabolites vary by sex and age, whereas other metabolites, particularly SCFAs, exhibit more context-dependent differences [11,12,13]. These inconsistencies highlight the need to incorporate sex as a biological variable when interpreting microbiota-derived metabolic signatures across the lifespan.

Despite the rapid advance in this field, most studies examining diet and the gut microbiota exclude sex as a variable or fail to analyze outcomes by sex [14,15]. This gap limits our understanding of how dietary interventions may exert sex-specific effects throughout life. Furthermore, older adults, a population in which both diet and microbiota exert a profound physiological influence, remain underrepresented in clinical and translational research [2,16]. Addressing these limitations is essential for advancing precision-nutrition approaches that consider both sex and microbiome variability.

In this review, we summarize current evidence on how diet and biological sex interact through the gut microbiota to shape ageing trajectories. First, we describe age-related changes in gut microbial composition. We then examine microbiota-derived metabolites and their relevance for sex-dependent metabolic and immunological pathways. Next, we review dietary patterns and functional foods that may exert sex-specific effects through microbiota modulation. Finally, we highlight methodological gaps and discuss how incorporating sex as a biological variable can provide personalized nutrition-based strategies to support healthy ageing.

## 2. Sex-Specific Determinants of Age-Related Disease

By 2050, one in six people worldwide will be over 65, marking an unprecedented demographic shift with profound social and economic implications [17]. Although life expectancy continues to rise, advances in healthy ageing have not kept pace, leaving older adults disproportionately affected by chronic disease, frailty, and dependency [1]. This gap is especially pronounced in developing regions, where rapid demographic transitions coexist with persistent socioeconomic inequalities [18]. Understanding the biological and environmental determinants that distinguish resilient from vulnerable ageing trajectories is therefore essential.

At the biological level, ageing is characterized by a progressive loss of systemic homeostasis driven by molecular damage, genomic instability, mitochondrial dysfunction, cellular senescence, and persistent low-grade inflammation, which together constitute the recognized hallmarks of ageing [19,20,21,22]. These interconnected processes contribute to major non-communicable diseases in later life, including cardiovascular disorders, osteoporosis, sarcopenia, type 2 diabetes, and neurodegeneration [23,24,25,26,27].

Importantly, the onset, progression, and clinical expression of these conditions differ markedly between men and women, positioning biological sex as a critical determinant of ageing trajectories [28]. Metabolically, men tend to develop type 2 diabetes at an earlier age and with a lower body mass index, whereas women present a higher burden of cardiometabolic risk factors at the time of diagnosis, such as obesity, hypertension, and dyslipidemia [29]. The abrupt loss of estrogen at menopause promotes visceral adiposity, insulin resistance, and dyslipidemia, diminishing the cardiometabolic advantage observed in premenopausal women [30,31]. In the skeletal system, estrogen deprivation accelerates bone resorption and increases trabecular fragility, representing a model of accelerated ageing in women compared to the gradual hormonal decline observed in men [32]. Cardiovascular ageing also diverges; men exhibit a higher atherosclerotic burden and increased rates of major cardiovascular events in midlife, whereas women more commonly develop heart failure with preserved ejection fraction at older ages, which is associated with microvascular dysfunction and myocardial stiffness [33,34]. Finally, neuronal markers such as circulating Neurofilament Light chain (NfL) increase with age and show modest but consistent sex differences, with higher concentrations in men, underscoring the need for sex-specific reference ranges when assessing neurodegeneration and cognitive decline [35].

Collectively, these findings demonstrate that hormonal, metabolic, immune, and microvascular mechanisms involved in ageing operate differently in men and women. These sex-specific vulnerabilities must be considered when developing strategies to prevent and manage chronic diseases associated with ageing.

## 3. Gut Microbiota Across Ageing

The ageing process is increasingly recognized as a phenomenon modulated by the gut microbiota, an ecosystem that integrates dietary, metabolic, and immune factors. This complex community, composed mainly of bacteria but also archaea, viruses, and fungi, resides primarily in the gastrointestinal tract [36]. Under eubiotic conditions, the adult gut microbiota is dominated by Firmicutes and Bacteroidetes, followed by Actinobacteria, Proteobacteria, and Verrucomicrobiota, and it exhibits high stability and metabolic versatility [37,38].

During ageing, however, this equilibrium gradually deteriorates gut microbiota diversity, the Firmicutes/Bacteroidetes ratio shifts, and Proteobacteria expand at the expense of beneficial genus such as *Lactobacillus* and *Bifidobacterium* [39]. Furthermore, the gut microbiota becomes increasingly individualized, with a loss of core taxa shared across older populations, reflecting ecological instability and functional deterioration [40]. The gut microbiota contributes to host physiology by fermenting complex carbohydrates and dietary fibers, thus supporting nutrient absorption, epithelial homeostasis, and energy balance [41]. It also participates in the biosynthesis and transformation of bioactive molecules involved in cellular renewal, metabolic regulation, and neural communication [42,43,44]. Concurrently, the gut microbiota modulates dietary and xenobiotic metabolism, influencing host detoxification and specific metabolic pathways [45,46,47].

As immune function weakens with age, microbiota–immune interactions become crucial for maintaining tolerance and restraining chronic inflammation [48]. Through these interconnected functions, microbial changes extend systemically to the gut–brain axis, energy balance, and behavior [49,50]. Accumulating evidence links these age-related alterations in microbial composition and activity with increased susceptibility to frailty, cognitive decline, and cardiovascular dysfunction [39,51,52,53]. Hormonal changes, particularly estrogen decline during menopause, further modulate microbial composition and metabolism [54]. Collectively, these shifts mark the transition from a stable, symbiotic ecosystem to an age-associated dysbiosis that underlies the metabolic and inflammatory mechanisms.

### Sex-Linked Features of the Gut Microbiota in Older Adults

In older adults, ageing is accompanied by a progressive restructuring of the gut microbiota, rather than a uniform loss of diversity. Comparative studies show that elderly and centenarian populations display distinct microbial configurations, characterized by a decline in butyrate producers such *Faecalibacterium prausnitzii* and *Roseburia* spp., alongside an expansion of opportunistic Proteobacteria [27]. However, extreme longevity appears to involve compensatory microbial reorganization, first described in studies of centenarians and later confirmed by functional metagenomic analyses, including the enrichment of metabolically active and anti-inflammatory taxa such as *Akkermansia muciniphila*, Christensenellaceae, *Bacteroides* spp., and *Lactobacillus* spp. [27,55,56]. This pattern suggests that ageing microbiomes may evolve towards frailty-associated dysbiosis or, alternatively, towards resilient states that preserve host–microbe homeostasis [57].

Ageing is also marked by increasing interindividual heterogeneity of the gut microbiota, changes in classical α-diversity are modest or context-dependent, and age-related shift in gut community composition may mask sex effects in later life [40,58]. Evidence from large human cohorts, including the Pinggu metagenomic project and European population datasets, indicates that women in early adulthood typically exhibit greater microbial diversity and an enrichment of health-associated taxa such as *Akkermansia muciniphila* and SCFA-producing Firmicutes, but these differences diminish with advancing age and the hormonal transition of menopause [59]. Cross-sectional analyses conducted from Latin American and European populations similarly show that sexual dimorphism peaks in young adulthood and converges by the seventh decade of life [58].

Nonetheless, compositional biases persist in older adults: women tend to maintain a higher relative abundance of Actinobacteria (particularly *Bifidobacterium*) and Firmicutes (e.g., *Blautia*, Lachnospiraceae), whereas men display an enrichment of Bacteroidota, taxa that in some contexts of uncontrolled expansion, has been associated with proinflammatory profiles [60]. Recent metagenomic analyses in centenarians from Hainan also support persistent sex differences: male centenarians showed higher α-diversity and enrichment of *Lactobacillus gasseri*, *L. oris*, and *L. salivarius*—species associated with antioxidant activity and immune tolerance—but also carried potentially pathogenic species such as *Clostridium hathewayi* and *Eggerthella lenta*. In contrast, female centenarians exhibited an enrichment in SCFA-producing species including *Prevotella copri*, *Roseburia inulinivorans*, and *Eubacterium rectale*, suggesting sex-specific microbial networks underlying distinct trajectories of healthy aging [61]. Sex-related differences also emerge in muscle physiology; in a cohort of elderly Koreans, microbial diversity and the presence of *Roseburia faecis* were correlated positively with skeletal muscle mass in men but not in women, suggesting a sex-dependent gut–muscle axis relevant to sarcopenia [62].

Across studies, inconsistencies arise due to differences in cohort composition, regional dietary patterns, medication use, and analytical methods, factors that often overshadow subtle sex effects in older adults. Furthermore, most human evidence is cross-sectional and therefore correlational. Mechanistic insights are derived primarily from animal studies demonstrating causal relationships between microbial composition, microbiota-derived metabolites, and systemic inflammation, which are discussed in later sections.

## 4. Inflammaging as a Microbiota–Immunosenescense Axis

Inflammaging is defined as the chronic low-grade inflammation that accompanies ageing, is a major driver of physiological decline, and increase disease susceptibility in later life [63]. This process arises from the convergence of the immune, cellular, and microbial factors, including intestinal microbiota dysbiosis and epithelial barrier dysfunction, that progressively contribute to cellular and immunological senescence [64].

**Figure 1:**
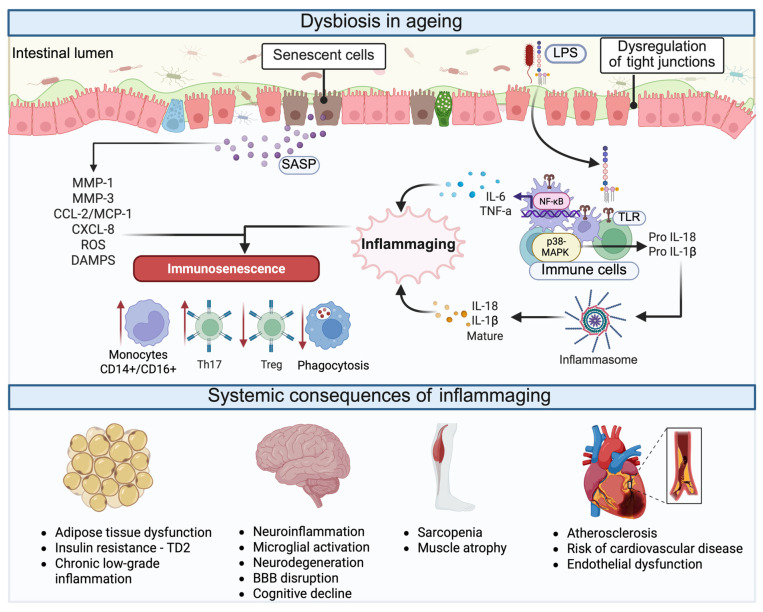
Dysbiosis, inflammaging, and systemic consequences in ageing. Age-related microbial imbalance and epithelial barrier dysfunction increase lipopolysaccharide (LPS) translocation and Toll-like receptor (TLR) activation, promoting NF-κB and p38-MAPK signaling and the secretion of pro-inflammatory cytokines (IL-6, TNF-α) and inflammasome-dependent mediators (IL-1β, IL-18). In parallel, senescent epithelial cells release cytokines, chemokines, matrix metalloproteinases, and reactive oxygen species through the senescence-associated secretory phenotype (SASP), reinforcing immunosenescence with expansion of CD14^+^/CD16^+^ monocytes and imbalance of Th17/Treg populations. These pathways sustain a chronic inflammatory state that drives metabolic, neural, muscular, and cardiovascular dysfunction characteristic of ageing. Red arrows indicate inflammatory progression, whereas black arrows indicate mechanistic pathways or causal interactions. Abbreviations: LPS, lipopolysaccharides; SASP, senescence-associated secretory phenotype; ROS, reactive oxygen species; DAMPs, damage-associated molecular patterns; NF-κB, nuclear factor kappa B; TLR, Toll-like receptor; BBB, blood–brain barrier. Created in BioRender. Hernandez, J. (2025) https://BioRender.com/2wy541c.

At the intestinal level, dysbiosis and epithelial barrier dysfunction increase intestinal permeability and facilitate the translocation of microbial products such as lipopolysaccharides (LPS) into the lamina propria. Subsequently, LPS binding to TLR-4 activates pro-inflammatory signaling cascades mediated by Nuclear Factor kappa-light-chain-enhancer of activated B cells (NF-κB) and Mitogen-Activated Protein Kinase-p38 (p38-MAPK) [65] (Figure 1). This activation promotes the sustained expression of pro-inflammatory cytokines, such as interleukin-6 (IL-6) and Tumor Necrosis Factor-alpha (TNF-α), together with NOD-like Receptor Family, Pyrin Domain Containing 3 inflammasome (NLRP3 inflammasome) activation, which stimulates the secretion of IL-1β and IL-18, thereby amplifying systemic inflammation [66]. Nevertheless, the precise contribution of LPS-driven signaling to systemic inflammaging remains uncertain, as it is influenced by host genetics, dietary patterns, and sex-dependent differences that collectively modulate gut microbiota composition and inflammatory response [25,66,67]. Dietary patterns strongly influence gut microbial ecology; for instance, fiber-rich diets favor Bacteroidetes and short-chain fatty acid production with anti-inflammatory effects, whereas high-fat Western diets reduce diversity and promote proinflammatory taxa such as Enterobacteriaceae [68,69,70]. These well-established links between diet, microbiota, and inflammation reinforce the need for integrative approaches combining microbial, metabolic, and immunological perspectives (Figure 1).

In parallel, and converging mechanistically with LPS-driven inflammation, the accumulation of senescent cells contributes to the same proinflammatory milieu through the Senescence-Associated Secretory Phenotype (SASP). Unlike the chronic immune activation triggered by LPS, SASP represents a chronic, cell-intrinsic source of inflammation that accumulates with age [48,71]. This phenotype is characterized by the release of inflammatory mediators (IL-6 and C-C motif chemokine ligand 1 (CCL-1)), chemokines (CXCL-8), matrix metalloproteinases (MMP-1, MMP-3), reactive oxygen species (ROS), and Damage-Associated Molecular Patterns (DAMPS), all of which reinforce the chronic inflammatory milieu [72,73]. Together, LPS-driven immune activation and SASP-derived mediators establish a self-perpetuating inflammatory loop. This vicious cycle promotes immunosenescense, marked by the expansion of pro-inflammatory CD14+/CD16+ monocytes [74] and an imbalance in Th17/Treg lymphocyte populations [75], both of which contribute to reduced phagocytic capacity and, ultimately, to a dysfunctional immune response unable to effectively resolve inflammation. These interconnected mechanisms, linking microbial dysbiosis, SASP-driven inflammation, and immune dysregulation, are summarized in Figure 1.

### Sex-Specific Features of Inflammaging

Inflammaging exhibits clear sexual dimorphism driven by hormonal, immune, and microbial differences. Women show stronger innate and adaptive immune responses across adulthood, with higher CD4^+^ T-cell counts and CD4:CD8 ratios [76], but this advantage diminishes after menopause, when the decline in estrogen reduces IL-10 and increases IL-6 and TNF-α, shifting the immune milieu towards inflammation [67]. Men exhibit earlier and more pronounced immunosenescence, marked by reduced naïve T cells and higher basal IL-6, TNF-α, and C-reactive Protein (CRP) levels, contributing to increased cardiometabolic vulnerability [23,76].

Microbiota–sex interactions further increase these pathways due to estrogen’s support of epithelial integrity, SCFA production, and immune tolerance [77,78], whereas their decline increases gut permeability and microglial reactivity, enhancing systemic and neuroinflammatory responses in older women [79]. Altogether, inflammageing arises from reciprocal interactions between microbial dysbiosis, epithelial barrier decline, immune remodeling, and SASP accumulation [67,80,81]. These pathways appear to be modulated by sex hormones and microbial metabolites, which shape different inflammatory outcomes in men and women. Since diet is a major regulator of gut microbiota composition, leading to SCFA production and epithelial permeability, nutritional strategies that modulate these pathways, such as fiber enrichment, SCFA-enhancing interventions, or the reduction of pro-inflammatory dietary patterns, offer sex-dependent opportunities to attenuate inflammaging and support healthy ageing.

## 5. Diet–Microbiota Interactions in Ageing

The interaction between diet and the gut microbiota constitutes a central axis regulating the physiological and pathological processes of ageing. Dietary patterns strongly shape microbial ecology throughout adulthood and later life, and age-related changes in food intake, such as reduced fiber consumption, lower dietary diversity, and higher intake of processed foods further contribute to microbial instability in older adults [82,83]. Specific dietary components, including fermentable fiber [84], polyphenols [85], omega-3 fatty acids [86], and plant-based protein sources associated with lower all-cause mortality [87], act as functional modulators of the gut microbiota by promoting beneficial taxa, suppressing pro-inflammatory species, and enhancing the production of beneficial metabolites [88].

Experimental evidence demonstrates that microbiota modulation can attenuate hallmarks of age-related decline. Fecal microbiota transplantation (FMT) from young to aged mice restores SCFA production, improves intestinal homeostasis, and enhances cognitive and locomotor performance [89]. Similarly, supplementation with bacteria such as *Akkermansia muciniphila* [90] or *Clostridium butyricum* [91], as well as microbial metabolites such as urolithin A, reduces systemic inflammation and oxidative stress and promotes functional improvements in ageing models [92]. Multi-strain probiotics containing *Lactobacillus* and *Bifidobacterium* spp. have also been shown to increase microbial diversity, reduce endotoxaemia, and improve markers of inflammation and frailty [93,94]. Observational and mechanistic studies in both experimental models and long-lived human populations consistently associate a diverse microbiota enriched in taxa such as *Akkermansia*, Christensenellaceae, and *Clostridium cluster* XIVa with reduced inflammation, enhanced immune function, and greater resilience to age-related deterioration [95,96].

These findings position diet as a key determinant of microbial composition and function during ageing. To clarify these mechanisms, we first outline the major microbiota-derived metabolites through which diet exerts systemic effects, followed by an examination of how specific dietary components such as fermentable fiber, polyphenols, omega-3 fatty acids, secondary bile acids, and protein sources modulate these metabolic pathways, leading to sex-specific healthy ageing.

### 5.1. Microbiota-Derived Metabolites as Mediators of Dietary Effects in Ageing

Microbiota-derived metabolites constitute a primary interface through which diet influences host physiology across lifespan. Among these, short-chain fatty acids (SCFAs), including acetate, propionate, and butyrate, play central roles in maintaining epithelial barrier integrity, regulating immune and metabolic homeostasis. Their decline with advancing age has been implicated in inflammaging, impaired energy balance, and increased vulnerability to chronic diseases [97].

Polyamines such as spermidine, spermine, and putrescine are another class of metabolites whose levels depend on both dietary precursors and microbial synthesis. These molecules support autophagy, DNA stability, intestinal barrier function, and immune regulation, yet their abundance decreases with ageing, potentially contributing to tissue dysfunction and reduced resilience to age-related diseases [98].

Other metabolites, including indole derivatives coming from tryptophan metabolism, secondary bile acids, and urolithins, exert neuromodulatory activity, as well as antioxidant and anti-inflammatory effects relevant to ageing biology [12,92,99]. In contrast, trimethylamine N-oxide (TMAO), generated from microbial metabolism of choline and carnitine, has been associated with endothelial dysfunction, cardiometabolic risk, and neuroinflammation in older adults [100,101], illustrating how diet–microbiota interactions may also contribute to pathological ageing.

Since the production of these metabolites depends strongly on dietary substrates and microbial composition, the following sections will describe how specific dietary components modulate these pathways and the ageing responses between men and women.

### 5.2. Fermentable Fiber and SCFA Production

The age-related decline in SCFAs is exacerbated by the low intake of soluble fiber, found in foods such oats, barley, legumes, green banana, asparagus, artichoke, and flaxseed, which limits the availability of fermentable substrates for commensal colonic bacteria [102,103]. Several studies show that fermentable fiber promotes the growth and metabolic activity of species such as *Faecalibacterium prausnitzii*, *Roseburia* spp., and *Bifidobacterium* [104], whose abundance decreases with age but whose activity is essential for the synthesis of SCFAs with anti-inflammatory properties [105]. In older Japanese adults, higher soluble fiber intake was linked to a greater relative abundance of butyrate-producing bacteria, and its sustained consumption contributed to the long-term maintenance of a stable, anti-inflammatory colonic microbiota [106], supporting the role of dietary fiber counteracting age-driven microbial instability.

**Figure 2:**
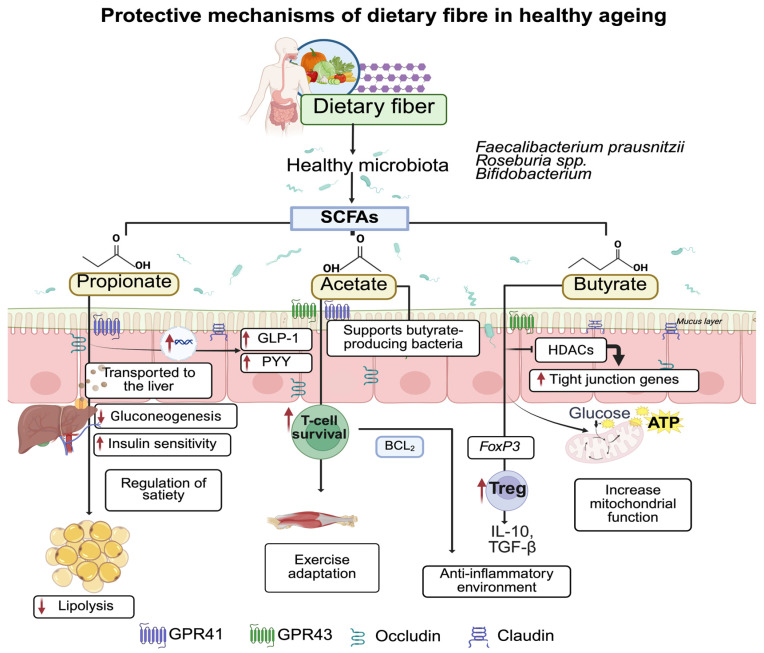
Protective mechanisms of dietary fiber–microbiota interactions in healthy ageing. Dietary fiber promotes the growth of beneficial bacteria such as *Faecalibacterium prausnitzii*, *Roseburia* spp., and *Bifidobacterium*, which produce short-chain fatty acids (SCFAs) with complementary metabolic and immunological effects. Propionate stimulates gut hormones GLP-1 and PYY, improving insulin sensitivity and satiety while reducing lipolysis. Acetate, the most abundant SCFA, supports butyrate-producing bacteria, fuels peripheral tissues, activates GPR43 to promote T-cell survival through BCL_2_ signaling, and enhances exercise adaptation and mitochondrial function. Butyrate strengthens epithelial integrity by upregulating tight-junction genes and HDACs and promotes anti-inflammatory Treg differentiation via FoxP3, IL-10, and TGF-β. Together, these mechanisms maintain intestinal barrier integrity, immune tolerance, and metabolic resilience, counteracting inflammaging and supporting healthy ageing. Abbreviations: SCFA, short-chain fatty acid; GLP-1, glucagon-like peptide 1; PYY, peptide YY; GPR, G-protein-coupled receptor; HDAC, histone deacetylase; Treg, regulatory T cell; IL-10, interleukin 10; TGF-β, transforming growth factor beta; ATP, adenosine triphosphate; BCL_2_, B-cell lymphoma 2. Created in BioRender. Hernandez, J. (2025) https://BioRender.com/ba5mc57.

Among SCFAs, butyrate plays a pivotal role in intestinal health. Butyrate supplementation enhances epithelial barrier integrity by upregulating tight-junction proteins such as claudin-1 and zonula occludens-1 (ZO-1), thereby reducing paracellular permeability and preventing bacterial translocation [41,107,108]. These effects are mediated, at least in part, by activation of G-protein-coupled receptor-43 (GPR43) in the cecal epithelium, as observed in animal models fed high-fat diets [109]. Butyrate also serves as the primary energy substrate for colonocytes and exerts robust epigenetic regulation via histone deacetylases (HDACs) inhibition [110], promoting FOXP3 and IL-10 expression and driving regulatory T cell lymphocyte (Treg) differentiation [111], a key mechanism in maintaining intestinal immune tolerance and mitigating immunosenescence (Figure 2).

Propionate and butyrate send signals through GPR43 and GPR41 [112], but propionate exerts distinct systemic effects. After colonic absorption and hepatic transport, propionate suppresses gluconeogenesis [113], improves insulin sensitivity [114], and modulates lipid metabolism, thereby contributing to cardiometabolic protection [115]. Clinical trials show that colonic infusion of propionate and acetate reduces plasma lipolysis [116], and that targeted colonic delivery of propionate stimulates Glucagon-like peptide-1 (GLP-1) and Peptide tyrosine tyrosine (PYY) release, reducing appetite and adiposity [117] (Figure 2). Dietary fermentable fibers such as inulin consistently enhance colonic SCFAs production and reduce total and LDL-cholesterol [118]. Additionally, murine models demonstrate that propionate prevents age-associated vascular calcification through favorable remodeling of the gut microbiota [119]. These findings highlight the complementary roles of SCFAs in energy homeostasis, gut hormone regulation, and mucosal immunity.

Acetate, the most abundant SCFA in systemic circulation, supports cross-feeding interactions that stimulate butyrate-producing bacteria, strengthening the ecological stability of the colonic microbiota [120]. Notably, supplementation with acetate or acetate-producing bacteria such as *Akkermansia muciniphila* has been shown to reverse ageing-associated alterations, including chronic low-grade inflammation, hepatic dysfunction, and impaired intestinal barrier integrity [90]. Acetate also activates GPR43 on immune cells, modulating systemic inflammation and preserving immune homeostasis (Figure 2) [121].

In addition, acetate exerts direct effects on adaptive immunity by promoting T cell survival through enhanced α-tubulin acetylation and activating the CD30/Bcl-2 pathway, thereby protecting against lymphocyte apoptosis, a mechanism relevant to counteracting immunosenescence [122].

As a consequence, the age-related decline in SCFAs contributes to a pro-inflammatory and metabolically impaired intestinal environment, thereby increasing susceptibility to chronic disease. While the epithelial, metabolic, and immunoregulatory actions of SCFAs operate broadly across ageing, emerging evidence indicates that both SCFA and the microbial response to fermentable fiber may diverge by biological sex. These patterns have been observed mainly in non-human primates and rodent models, where ageing females exhibit greater vulnerability to SCFA declines and distinct microbiota-mediated responses compared with males. In contrast, human studies have demonstrated that higher fiber intake increases SCFAs; however, non-reproducible sex-specific differences in SCFA concentrations or responsiveness to fermentable fiber have been demonstrated. Preclinical and clinical evidence is shown in Table 1. Maintaining adequate soluble-fiber intake and supporting SCFA-producing microbial communities remain central nutritional strategies for healthy ageing, and future trials with sex-stratified analyses are needed to clarify how SCFA biology may differ between older men and women.

### 5.3. Polyphenols and Oxidative Stress

Polyphenols in fruits, vegetables, and legumes act as indirect prebiotics, as many are poorly absorbed in the small intestine. Once they reach the colon, they are metabolized by the gut microbiota through reactions such as glycoside hydrolysis, ring fission, and demethylation [128]. These reactions generate low-molecular-weight phenolic metabolites with greater bioavailability and biological activity, which explains their “prebiotic-like” effects on microbial ecology and host redox homeostasis [129,130].

**Figure 3:**
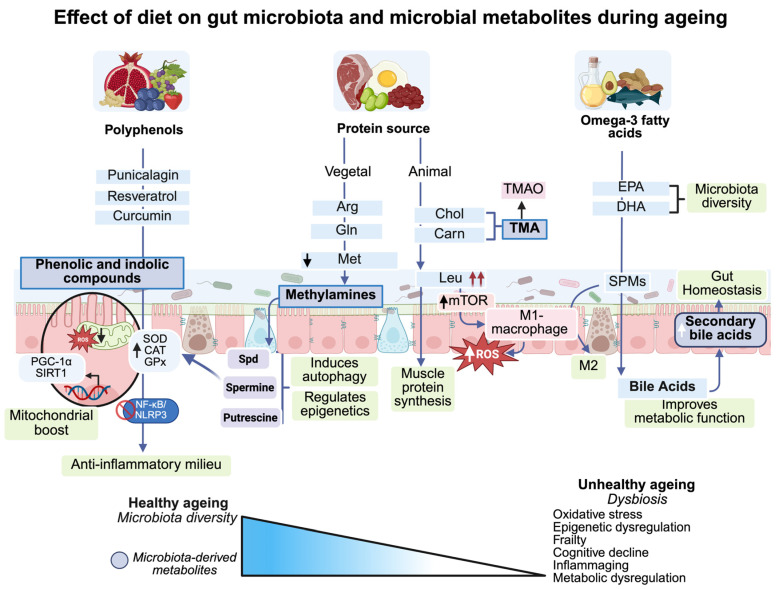
Impact of dietary components on microbiota-derived metabolites during ageing. Polyphenol-rich foods generate phenolic and indole compounds that enhance mitochondrial function (via SIRT1/PGC-1α) and antioxidant enzymes (SOD, CAT, GPx) while inhibiting inflammatory pathways (NF-κB, NLRP3-inflammasome), contributing to an anti-inflammatory milieu. Protein sources shape microbial metabolism: plant protein-derived amino acids such as Arg (arginine) and Gln (glutamine) favor production of short-chain fatty acids (SCFAs) and polyamines (Spd, spermidine; spermine, and putrescine), supporting autophagy and muscle maintenance. In contrast, Met (methionine), Leu (leucine), Chol (choline), and Carn (carnitine) from animal protein increase TMA (trimethylamine) and hepatic TMAO (trimethylamine-N-oxide), promoting ROS production and M1 macrophage activation. Omega-3 fatty acids (EPA, DHA) enhance microbial diversity and give rise to SMPs (specialized pro-resolving mediators) such as PD1 (protectin D1), RvD1 (resolving D1), and MaR1 (maresin 1), which drive M2 (pro-resolving) macrophage-mediated tissue repair; microbiota-derived secondary bile acids further support gut homeostasis and metabolic function. Created in BioRender. Hernandez, J. (2025) https://BioRender.com/g1friry.

Alongside these microbiota-dependent actions, polyphenols also exert direct antioxidant effects by scavenging reactive oxygen species (ROS), including superoxide and hydroxyl radicals, through electron or hydrogen donation [131,132]. They also activate endogenous antioxidant defenses such as superoxide dismutase (SOD), catalase, and glutathione peroxidase (GPx), whose activities decline with ageing [133] (Figure 3).

Several polyphenols also display targeted mitochondrial actions. For instance, resveratrol promotes mitochondrial biogenesis through Sirtuin 1 (SIRT1), and peroxisome proliferator-activated receptor gamma coactivator 1-alpha (PGC-1α) activation, thereby reducing mitochondrial ROS and improving mitochondrial function [45,134,135]. Similarly, pomegranate-derived ellagitannins (punicalagin, ellagic acid) enhance SIRT1 signaling, stabilize mitochondrial pathways, and attenuate inflammatory responses [136]. Other polyphenols act primarily through immunomodulatory pathways: curcumin inhibits NF-kB activation and downregulates pro-inflammatory cytokines expression [137], while epigallocatechin-3-gallate (EGCG) and gallic acid also suppress NF-kB/NLRP3 inflammasome signaling in experimental models [138]. Consistently, resveratrol and pomegranate-derived compounds reduce inflammaging by inhibiting NF-κB and NLRP3 activation and decreasing IL-1α and IL-18 expression [139].

Emerging technological approaches highlight the synergy between polyphenols and the gut microbiota. Polyphenol-based nanostructures used to coat probiotics or antioxidant nanozymes reduce intestinal inflammation, improve microbial composition, and decrease IL-6 and TNF-α expression in murine colitis models [140]. Resveratrol exemplifies this dual action: beyond activating SIRT1/PGC-1α and nuclear factor erythroid-2-related factor-2 (Nrf2) in host tissues, it modulates the gut microbiota and the tryptophan–kynurenine axis, reducing oxidative stress and inflammation; fecal transplantation experiments confirmed that these effects are partly microbiota-dependent [141]. Microbial metabolism of polyphenols further amplifies their anti-ageing effects, fermentation of berry anthocyanins by *Lactobacillus plantarum SC-5* yields phenolic acids that increase *Bifidobacterium* abundance, elevate SCFA levels, reduce ROS, and upregulate SIRT1 and brain-derived neurotrophic factor (BDNF) in D-galactose-induced ageing models, improving cognitive performance [142]. In addition, quinic acid from millet attenuates neuroinflammation and oxidative stress in high-fat-diet-induced ageing through microbial tryptophan metabolites, indole-3-acetic acid (IAA), and kynurenic acid (KYNA), which replicate the effects of quinic acid and suppress the DR3/IKK/NF-κB pathway, correlating with lower amyloid-β peptide and phosphorylated Tau protein (pTau) [130].

Complex food matrices also illustrate the microbiota–antioxidant synergy. In a D-galactose ageing model, red ginseng (rich in polyphenols and rare ginsenosides) produced stronger anti-ageing effects than white ginseng: reduced malondialdehyde (MDA), increased SOD and catalase, improved behavioral outcomes, suppressed NF-κB, caspase-3 and PI3K–Akt activity, and remodeled the gut microbiota by enriching *Bifidobacterium* and *Akkermansia* [143]. These findings highlight how embedding polyphenols within food matrices can potentiate both microbial modulation and antioxidant effects (Figure 3).

Although most of these mechanisms apply broadly to ageing physiology, the extent to which the metabolism and biological effects of polyphenols differ between men and women remains unclear. Preclinical models consistently demonstrate sex-dependent antioxidant and metabolic responses to polyphenols, including differential modulation of ROS, SOD/CAT/GPx activity and inflammatory pathways, but these patterns have not been reproduced in human studies. Notably, clinical trials in older adults rarely stratify outcomes by sex, and interindividual variability in microbial composition appears to overshadow potential sex effects, particularly in the production of urolithin and phenolic acid metabolites [144,145]. These emerging gaps are summarized in Table 1. The heterogeneity of polyphenol formulations, dosages, and food matrices further complicates comparisons across interventions, underscoring the need for rigorously designed, sex-stratified clinical studies to clarify the therapeutic and sex-specific potential of polyphenols in ageing populations.

### 5.4. Omega-3 and Anti-Inflammatory Effects

Long-chain omega-3 polyunsaturated fatty acids, primarily eicosapentaenoic acid (EPA) and docosahexaenoic acid (DHA), are recognized as key modulators of healthy ageing due to their anti-inflammatory and immunomodulatory properties [146]. These fatty acids incorporate into cell membranes, where they influence membrane fluidity, reorganize lipid microdomains, and modify immune receptor signaling, resulting in reduced NF-kB activation [147]. At the molecular level, EPA and DHA act as precursors of specialized pro-resolving mediators (SPMs) including resolvins, protectins, and maresins. Among these, Maresin-1 (MaR1) promotes macrophage polarization toward an anti-inflammatory M2 phenotype and suppresses NF-kB activity, mechanism predominantly demonstrated in preclinical models [148,149] (Figure 3).

The interaction between omega-3 fatty acids and the gut microbiota has emerged as an axis in ageing. Supplementation with EPA and DHA increases the abundance of *Akkermansia muciniphila* [150] and SCFA-associated genera such as *Lactobacillus* spp. and *Bifidobacterium* [151], taxa associated with improved epithelial barrier integrity, bile acid metabolism, and reduced low-grade inflammation. These microbiota shifts also influence the conversion of secondary bile acids, regulating insulin sensitivity, systemic inflammation, and innate immune signaling through receptors such as Farnesoid X receptor (FXR) and G protein-coupled bile acid receptor 1 (GPBAR1, also known as TGR5), processes particularly relevant in ageing [152].

Experimental ageing models provide further evidence for these gut–brain and gut–immune interactions. In D-galactose-induced accelerated ageing mice, omega-3 supplementation improved hippocampal-dependent memory, increased antioxidant enzymes (SOD, GPx and catalase), and shifted the gut microbiota toward an eubiotic configuration, with synergistic effects observed when combined with polyphenols and carotenoids [153]. In humans, higher omega-3 intake has been associated with preserved hippocampal volume, reduced cortical atrophy [154], and lower risk of depression in older adults [155].

Despite these promising findings, results from randomized controlled trials in older adults remain inconsistent. Variability in supplementation duration, EPA/DHA formulations, and baseline omega-6/omega-3 ratios contribute to heterogeneous outcomes [146]. Interindividual variation in metabolic and triglyceride responses to omega-3 supplementation further highlights the gut microbiota as a determinant of omega-3 efficacy [156]. Emerging evidence suggests that the metabolic handling, inflammatory responses, and microbial remodeling induced by omega-3 fatty acids may differ between men and women. Sex-dependent differences have been documented in omega-3 absorption, incorporation into phospholipid membranes, and conversion into SPMs, with women often showing greater EPA/DHA incorporation and higher increases in pro-resolving mediators. Preclinical models further reveal sex- and hormone-dependent remodeling of the gut microbiota in response to ω-3-rich diets [149,156]; however, such microbiota-mediated sex differences have not been demonstrated in older humans, largely due to the lack of sex-stratified analyses in clinical trials. These emerging patterns and evidence gaps are summarized in Table 1.

### 5.5. Proteins, Polyamines, and TMAO

The source and quality of dietary proteins are major modulators of ageing, influencing skeletal muscle anabolism, immune function, and gut microbiota metabolism [157]. Plant-derived proteins, typically richer in arginine and glutamine and naturally lower in methionine, promote an anti-inflammatory intestinal environment, particularly when consumed alongside fermentable fibers [158] (Figure 3). This dietary combination enhances microbial fermentation, increases SCFA production, and provides precursors for the synthesis of polyamines such as spermidine, spermine, and putrescine, whose levels decline with age and are essential for autophagy, DNA integrity, and immune regulation [159].

Mechanistic models reinforce these links. Spermidine supplementation improves intestinal barrier function, restores microbial diversity, and reduces systemic inflammation in diet-induced obesity models [160]. Spermine enhances DNA methylation in hepatic and colonic tissues, contributing to epigenetic stability and lifespan extension in rodents fed polyamine-rich diets [161] (Figure 3). Beyond classical polyamines, ergothioneine, an antioxidant amino acid derivative produced by fungi and bacteria and abundant in mushrooms and legumes, has been proposed as a “longevity vitamin” due to its capacity to reduce oxidative stress and confer neuroprotection [162,163]. Human studies support these observations. In the Korean Multi-Rural Communities Cohort, higher intake of soy protein and isoflavones was inversely associated with metabolic syndrome risk, particularly in women [164]. Similarly, data from the Nurses’ Health Study (*n* > 48,000) show that higher midlife plant protein intake predicts a greater likelihood of achieving healthy ageing over three decades of follow-up [165].

In contrast, excessive consumption of animal-derived proteins, typically richer in methionine, lysine, and leucine, may activate detrimental metabolic pathways. Methionine promotes oxidative stress via homocysteine generation [87], whereas methionine restriction (MR) extends lifespan and mitigates cognitive decline in experimental Alzheimer’s models, partly through microbiota-dependent increases in indole-3-propionic acid and activation of PPARα signaling [166]. Importantly, MR outcomes are not uniform; benefits vary by sex and age, with stronger responses observed in males and diminished effects when initiated in adulthood, underscoring sex-specific metabolic adaptation [167]. MR also induces metabolic reprogramming in liver and brain, enhancing fatty-acid oxidation and Fibroblast growth factor-21 (FGF21) secretion, potentially delaying steatosis and neurodegeneration [168]. Leucine further exemplifies the dual nature of amino acids in ageing: although essential for overcoming anabolic resistance and stimulating muscle protein synthesis in older adults [169], chronic leucine excess hyperactivates mTOR signaling, contributing to insulin resistance, chronic low-grade inflammation, and decreased longevity [163], Figure 3.

A distinct protein-related pathway relevant to ageing involves microbial metabolism of choline and carnitine. Diets high in these substrates promote bacterial production of trimethylamine (TMA), which is converted in the liver to trimethylamine-N-oxide (TMAO). Elevated circulating TMAO impairs endothelial function through oxidative stress in humans and mice [100] and has been implicated in sarcopenic obesity and neuroinflammation in naturally aged rats [170,171]. Notably, TMAO levels and cardiovascular susceptibility appear to differ by sex, with men generally displaying higher circulating TMAO and stronger associations with vascular and metabolic dysfunction as shown in Table 1.

Taken together, these findings highlight the contrasting effects of protein sources on gut microbiota composition, polyamine availability, and TMAO-related cardiometabolic pathways. Dietary patterns enriched in plant-based proteins, particularly when combined with fermentable fibers, appear most effective for supporting microbial diversity, enhancing SCFA and polyamine synthesis, and reducing TMAO-associated metabolic risk. Although preclinical models demonstrate clear sex-dependent responses to plant and animal protein, especially in methionine restriction outcomes and TMAO-related pathways, such sex-specific microbial and metabolic effects have not been consistently demonstrated in older humans, largely due to the scarcity of sex-stratified clinical analyses. These gaps underscore the need to integrate biological sex when designing and evaluating protein-based nutritional strategies in ageing populations.

### 5.6. Bile Acids as Systemic Modulators of Ageing

In addition to their role in lipid emulsification, bile acids (BAs) act as endocrine-like signaling molecules that regulate glucose homeostasis, lipid metabolism, thermogenesis, and energy expenditure through activation of nuclear and membrane receptors such as FXR and TGR5 [176]. Primary BAs, cholic acid (CA) and chenodeoxycholic acid (CDCA), are synthetized in the liver from cholesterol via the rate-limiting enzyme cholesterol 7α-hydroxylase (CYP7A1) [11]. After entering the intestine, they undergo microbial deconjugation and 7α-dehydroxylation by bile salt hydrolases and specialized bacteria taxa, generating the secondary BAs deoxycholic acid (DCA), lithocholic acid (LCA), and ursodeoxycholic acid (UDCA), which enhance the chemical diversity and signaling potency of the BA pool [177]. Through intestinal FXR activation, BAs stimulate fibroblast growth factor 15/19 (FGF15/19), which circulates to the liver and represses CYP7A1 transcription, establishing a tightly regulated microbiota–BA–liver feedback loop that maintains systemic homeostasis [178].

Ageing profoundly disrupts this system. In humans, cross sectional data from adults in the KarMeN study show that older individuals have higher fasting concentrations of conjugated primary BAs and altered BA pattern, including sex-specific differences in CDCA and its conjugates [7]. Combined with evidence from postprandial metabolic challenges, older adults also exhibit a shift toward a more hydrophilic, less diverse BA pool, partly driven by age-related modifications in the gut microbiome [179]. Gut dysbiosis reduces microbial deconjugation and secondary BA production, thereby modifying FXR/TGR5 signaling [12].

These alterations depend on biological sex; in both humans and rodent models, older females demonstrate increased intestinal absorption of conjugated primary BAs via upregulation of Apical Sodium-dependent Bile Acid Transporter (ASBT), resulting in elevated circulating and brain BA levels associated with synaptic loss and cognitive decline, and BA sequestration improves these phenotypes [172]. Ageing males, in contrast, exhibit distinct shifts in BA composition and hepatic transporter expression link to metabolic and neurodegenerative outcomes [174]. In humans, dysregulated cholesterol catabolism and altered circulating primary BAs are associated with dementia risk and cortical and white-matter changes, with sex-dependent patterns in BA receptor-related gene expression in the brain [173], these sex-differences are summarized in Table 1. Together, these findings indicate that microbiota-dependent conversion of primary BAs is not only essential for BA homeostasis but also a critical determinant of sex-specific metabolic, inflammatory, and cognitive outcomes across ageing.

## 6. Biological Sex as a Modulator of the Interplay Between Microbiota, Diet, and Ageing

The concept of the *microgenderome* describes the bidirectional interface between sex hormones and the gut microbiota, helping explain why men and women with comparable microbial taxa often display divergent physiological outcomes [4,180]. Ageing amplifies these differences through endocrine decline. In women, menopause-associated estrogen loss is accompanied by reduced abundance of anti-inflammatory taxa such as *Faecalibacterium prausnitzii* and *Akkermansia*, together with expansion of pro-inflammatory species a [181,182]. A central mechanism is the *estrobolome*, whose β-glucuronidase activity regulates enterohepatic estrogen cycling. Declines in estrobolome function are linked to osteoporosis, endothelial dysfunction, and cognitive decline [183,184].

In men, testosterone declines more gradually, but microbial metabolism directly influences androgen availability. *Clostridium scindens* and related taxa convert testosterone into less active metabolites, reducing its biological activity [185]. Clinical evidence shows that men with type 2 diabetes present concurrent low testosterone levels and altered microbial composition, suggesting a bidirectional axis [186]. Exogenous testosterone therapy in transgender adults also induces compositional shifts, such as enrichment of *Ruminococcus* and reduction of *Lactobacillus*, reinforcing a causal hormone–microbiome interaction [187,188,189]. Beyond baseline hormone levels, circadian oscillations of the microbiota synchronize with immune and metabolic pathways in a sex-dependent manner and are more disrupted in males exposed to obesogenic diets [188,189].

Mechanistically, sex hormones and the gut microbiota engage in a bidirectional regulatory loop that shapes ageing outcomes. Estrogens modulate intestinal permeability, immune signaling, and bile-acid turnover through ERα/ERβ pathways in epithelial and immune cells, altering microbial habitat and metabolic outputs [54]. Conversely, microbial β-glucuronidases and sulfatases regulate enterohepatic estrogen recycling, affecting systemic hormone availability and inflammatory response [184]. For androgens, the gut microbiota modifies steroid structure and influences androgen-responsive immune-metabolic pathways, while testosterone therapy induces predictable microbiota shifts in humans, strengthening evidence that hormonal perturbations directly reshape microbial community structure and function [187]. Together, these mechanisms support a unified microbiota–sex hormone axis, integrating steroid signaling, microbial metabolism, and dietary exposures into a coherent framework for sex-specific ageing.

Emerging evidence suggests that microbiome-derived biomarkers, including SCFA profiles, TMAO, phenylacetylglutamine, bile-acids, and estrobolome activity, may help to predict sex-specific ageing responses, although validation in humans remains limited. These biomarkers reflect integrated influences of sex hormones, immune response, and dietary exposures.

### 6.1. Sex-Specific Microbial Metabolite Profiles and Disease Risk

Gut-microbiota-derived metabolites shape distinct immunometabolic profiles across sexes. In women, postmenopausal estrogen loss coincides with reduced SCFAs (butyrate and propionate) and diminished tryptophan-derived indoles, impairing mitochondrial function and immune tolerance [190,191]. Reduced estrobolome activity further lowers systemic estrogens, heightening risks for osteoporosis, neurodegeneration, and autoimmune diseases [181,192].

Men, in contrast, accumulate higher levels of pro-atherogenic metabolites such as TMAO and phenylacetylglutamine, which rise with age and strongly predict vascular ageing and cardiometabolic dysfunction [193,194]. Finally, Hepatic transcriptomics confirm sex-specific remodeling of bile acids, with deoxycholic acid increasing with age in males but declining in females; these patterns are reversible after young-to-old microbiota transplantation [195,196]. Isoflavone metabolism is another example of dimorphism: the ability to convert daidzein into equol, a metabolite with estrogenic and anti-inflammatory activity, is more prevalent in women, particularly postmenopausal women, than in men [197,198]. In ovariectomized rats, *Lactobacillus intestinalis* supplementation increased equol and γ-aminobutyric acid (GABA), alleviating menopausal symptoms [125].

Collectively, these findings show that diet-derived compounds, once metabolized by the microbiota, generative protective or harmful metabolites in a sex-dependent way.

### 6.2. Sex-Dependent Dietary Responsiveness

Interventions targeting the microbiota display dimorphic outcomes. In a randomized trial in older adults, 12 weeks of probiotics reduced CD4+ T-cells and Firmicutes abundance in women, whereas in men, it decreased dendritic cells and Enterobacteriaceae [199]. In obesity, women respond more favorably to Mediterranean diets (showing lower high-sensitivity C-reactive protein (hs-CRP)), whereas men experience greater metabolic improvements with carbohydrate restriction [200,201]. In middle-aged obese mice, sleeve gastrectomy and intermittent fasting improved weight loss and inflammation more strongly in females than in males [202]. Fiber-based interventions also show sex specificity; in aged mice, inulin restored epithelial integrity and reduced inflammation, but metabolite profiles differed substantially by sex [203].

These sex-specific nutritional responses contribute to divergent disease risks; loss of SCFAs and indoles predisposes women to neuroinflammation and mitochondrial dysfunction, consistent with the higher burden of Alzheimer’s disease and autoimmune disorders [204,205,206]. In men, excessive TMAO production and bile acid dysregulation accelerate endothelial dysfunction and cardiovascular ageing [2,207].

Together, these findings support a unified microbiota–sex hormone axis, in which microbial metabolism, endocrine signaling (including estrogen and androgen pathways), and dietary exposures interact bidirectionally to shape sex-specific ageing outcomes. This framework integrates mechanistic evidence, from steroid metabolism and receptor signaling to metabolite-driven immunometabolism, clarifying how biological sex modulates the diet–microbiota–ageing interface.

### 6.3. Current Challenges and Future Perspectives

Despite growing evidence that biological sex influences the microbiota–diet–ageing axis, significant knowledge gaps remain. Most mechanistic insights arise from preclinical models in which hormonal status and dietary exposures can be experimentally controlled, whereas human studies are predominantly observational and often lack sex-stratified or hormone-stratified analyses. As a result, key processes such as estrobolome activity, androgen metabolism, and microbial contributions to steroid signaling are well described in experimental systems [183,184] but remain largely inferential in clinical populations [181,182].

Similar limitations apply to nutritional interventions. Preclinical studies consistently demonstrate sex-dependent responses to dietary fiber, methionine restriction, intermittent fasting, and probiotics [202,203,208]; however, clinical trials in older adults rarely incorporate sex or endocrine status as primary analytical variables. This limits the ability to determine whether dietary strategies exert distinct microbiota-mediated effects in ageing women versus men.

It is necessary to validate functional microbiome biomarkers, such as SCFA profiles, bile-acid signatures, TMAO, indole derivatives, equol phenotype, and estrobolome activity, for predicting sex-specific ageing outcomes. Although these candidates show promise, longitudinal human data integrating hormone levels, dietary patterns, and microbiome function are limited.

Future research should prioritize study designs that evaluate sex, age, and diet as interacting variables rather than isolated factors. This includes standardized microbiome pipelines, endocrine profiling, and multi-omics approaches to clarify causal mechanisms. Such strategies will be essential for translating mechanistic insights into sex-informed dietary recommendations aimed at promoting healthy ageing.

## 7. Conclusions

This review underscores the central role of the diet–microbiota axis in shaping ageing outcomes and highlights that biological sex critically modifies metabolic, inflammatory, and microbial responses throughout later life. While mechanistic studies reveal robust sex-dependent pathways (such as differences in SCFA decline, estrobolome activity, TMAO production, and bile-acid remodeling), human evidence remains limited by the scarcity of sex-stratified and hormone-informed analyses.

From a precision-nutrition perspective, current findings support several implications. Postmenopausal women, who experience reduced estrobolome activity and SCFA production, may particularly benefit from high-fiber dietary patterns, plant-based protein sources, and polyphenol-rich foods, which collectively enhance microbial diversity, promote butyrate producers, and mitigate inflammatory decline. Older men, who typically show higher circulating TMAO and PAGln, may benefit from reducing red meat and egg-derived choline-rich foods, increasing omega-3 intake, and emphasizing fermentable-fiber sources that counteract proteolytic fermentation and cardiometabolic risk.

At the population level, these findings point to the need for sex-specific dietary recommendations that integrate microbiome function, endocrine status, and metabolic phenotype. Translational progress, however, requires large, longitudinal, and rigorously sex-stratified clinical trials, incorporation of functional microbiome biomarkers, and mechanistic evaluation of the microbiota–sex hormone axis. Such approaches will be essential for developing personalized nutritional strategies that effectively support healthy ageing and reduce sex-specific disease vulnerabilities.

## Figures and Tables

**Table 1:** Sex-specific effects of microbiota-derived metabolites during ageing.

Dietary Component/Intervention	Category	Main Microbiota/Metabolite Changes	Population/Model	Sex-Specific Findings	Refs.
Fermentable fiber (inulin, GOS, resistant starch, arabinoxylans)	Prebiotic	↑ *Bifidobacterium*, ↑ *Lactobacillus*, ↑ *Akkermansia*, ↑ SCFAs (acetate, propionate, butyrate), ↓ Proteolytic metabolites (p-cresol, phenols). Ageing models: ↑ butyrate-producers, ↑*Faecalibaculum*, ↑ *Parabacteroides.*	Human RCTs in middle-aged and older adults *Macaca mulatta* (adult-aged) Ovariectomized rats Aged APOE4 mice	Female macaques show greater propionate decline with ageing; ApoE4 females respond more strongly to inulin (↑ diversity, ↓ inflammation); estrogen-deficient rats show ↓ butyrate. No consistent human sex-effects.	[106,118,123,124,125,126,127]
Polyphenols (flavonoids, ellagitannins, phenolic acids, anthocyanins, catechins)	Prebiotic-like	Microbial conversion to urolithins and phenolic acids. ↑ SCFAs, ↑ *Bifidobacterium*, ↑ *Lactobacillus*, ↑ *Akkermansia*, ↓ Inflammatory taxa	Older adults; aged mice and rats; human interventions with ellagitannin-rich foods.	Rodent models show sex-dependent antioxidant and metabolic responses to polyphenols. In humans, urolithin phenotypes reflect microbiota structure but show no consistent sex differences; most trials lack sex-stratified analyses.	[45,85,128,129,130,131,132,133,134,135,136,137,138,139,140,141,142,143,144,145]
Omega-3 fatty acids (EPA/DHA)	Nutritional supplement	↑ *Akkermansia,* ↑ *Lactobacillus*, ↑ *Bifidobacterium,* ↑ SCFA BA remodeling via FXR/TGR5. ↑ SPMs: Resolvins, Protectins, Maresins	Older adults; D-galactose-induced ageing mice; Alzheimer models; hormone status and omega-6/omega-3 ratio studies	Women show greater EPA/DHA incorporation and SPM increased; stronger microbiota shifts in ageing females (rodent models). Men: distinct BA/SCFA responses; omega-3 effects modulated by androgen status. Humans: metabolic sex differences present, but microbiota-specific ageing effects are unclear due to limited sex-stratified data.	[146,147,148,149,150,151,152,153,154,155,156]
Plant-based protein (legumes, soy, pulses, whole gains)	Protein source/prebiotic-like	↑ *Bifidobacterium,* ↑ *Lactobacillus,* ↑ SCFAs, Microbial conversion of isoflavones to equol and phenolics, ↓ Proteolytic metabolites	Human observational and intervention studies;Ageing rodent models	Women show higher prevalence of equol-producer phenotype; SCFA responses often stronger in females. Men display more variable microbial/metabolic responses. Human data limited.	[157,158,159,160,161,162,163,164,165,166,167,168,169,170,171]
Animal protein (red meat, poultry, eggs, dairy)	Protein source	↑ TMA, ↑ Proteolytic metabolites, ↑ *Clostridium* spp, ↑ *Desulfovibrio,* ↑PAGln	Human cohorts; Controlled feeding studies; Ageing rodents	Men typically show higher TMAO/PAGln due to microbiota composition and lower estrogenic modulation; postmenopausal women show increased TMAO with estrogen decline. No human trials assessing sex-specific ageing effects.	
Microbiota–bile acid interactions (microbial deconjugation, 7α-dehydroxylation, ASBT/FXR/TGR5 signaling)	Microbial metabolite pathway	Human: ↑ TCA, ↑ GCA, ↓ DCA, LCA, UDCA; altered fasting/postprandial BA patterns with age. Aging models: ↑ ASBT, ↓ Microbial 7α-dehydroxylation; BA imbalance restores with microbiota remodeling.	Healthy adults (18–80 years) from KarMeN cohort; Older adults with metabolic tests; Ageing cognitive cohorts; Aged models	Older women: higher conjugated BAs and greater BA absorption. Ageing men: BA composition shifts and altered receptor expression. Rodent ageing: females show greater BA absorption dysregulation; males show altered BA transporters and Cyp7a1. Humans: sex-specific fasting BA profiles, with older women showing higher conjugated BAs	[7,172,173,174]

Abbreviations: APOE4, apolipoprotein E ε4 allele; ASBT, apical sodium-dependent bile acid transporter; BA, bile acid; CA, cholic acid; CDCA, chenodeoxycholic acid; CYP7A1, cholesterol 7α-hydroxylase; DCA, deoxycholic acid; DHA, docosahexaenoic acid; EPA, eicosapentaenoic acid; FGF15/19, fibroblast growth factor 15/19; FXR, farnesoid X receptor; GOS, galactooligosaccharides; LCA, lithocholic acid; PAGln, phenylacetylglutamine; RCT, randomized controlled trial; ROS, reactive oxygen species; SCFAs, short-chain fatty acids; SPMs, specialized pro-resolving mediators; TGR5, Takeda G protein-coupled bile acid receptor 5; TMA, trimethylamine; TMAO, trimethylamine N-oxide; UDCA, ursodeoxycholic acid. Arrows indicate direction of change: ↑ increase; ↓ decrease. In skeletal muscle, acetate improves mitochondrial function, supports exercise adaptation, and contributes to the prevention of sarcopenia [175]. These interconnected processes relevant to ageing are summarized in Figure 2 and Figure 3.

## Data Availability

No new data were created or analyzed in this study. Data sharing is not applicable to this article.
